# The Length of Vesicular Stomatitis Virus Particles Dictates a Need for Actin Assembly during Clathrin-Dependent Endocytosis

**DOI:** 10.1371/journal.ppat.1001127

**Published:** 2010-09-30

**Authors:** David K. Cureton, Ramiro H. Massol, Sean P. J. Whelan, Tomas Kirchhausen

**Affiliations:** 1 Department of Cell Biology, Harvard Medical School, and Immune Disease Institute at Children's Hospital, Boston, Massachusetts, United States of America; 2 The Division of Gastroenterology and Nutrition, Children's Hospital, Boston, Massachusetts, United States of America; 3 Department of Microbiology and Molecular Genetics, Harvard Medical School, Boston, Massachusetts, United States of America; The Salk Institute for Biological Studies, United States of America

## Abstract

Microbial pathogens exploit the clathrin endocytic machinery to enter host cells. Vesicular stomatitis virus (VSV), an enveloped virus with bullet-shaped virions that measure 70×200 nm, enters cells by clathrin-dependent endocytosis. We showed previously that VSV particles exceed the capacity of typical clathrin-coated vesicles and instead enter through endocytic carriers that acquire a partial clathrin coat and require local actin filament assembly to complete vesicle budding and internalization. To understand why the actin system is required for VSV uptake, we compared the internalization mechanisms of VSV and its shorter (75 nm long) defective interfering particle, DI-T. By imaging the uptake of individual particles into live cells, we found that, as with parental virions, DI-T enters *via* the clathrin endocytic pathway. Unlike VSV, DI-T internalization occurs through complete clathrin-coated vesicles and does not require actin polymerization. Since VSV and DI-T particles display similar surface densities of the same attachment glycoprotein, we conclude that the physical properties of the particle dictate whether a virus-containing clathrin pit engages the actin system. We suggest that the elongated shape of a VSV particle prevents full enclosure by the clathrin coat and that stalling of coat assembly triggers recruitment of the actin machinery to finish the internalization process. Since some enveloped viruses have pleomorphic particle shapes and sizes, our work suggests that they may use altered modes of endocytic uptake. More generally, our findings show the importance of cargo geometry for specifying cellular entry modes, even when the receptor recognition properties of a ligand are maintained.

## Author Summary

We present a detailed comparison between the clathrin-dependent entry mechanisms of a parental virus (VSV) and its smaller defective interfering particle (DI-T). We used the difference in virion length to probe why actin assembly is required for the uptake of full-length VSV particles by nonpolarized mammalian cells. By imaging the entry of single particles in an unbiased manner, we resolved differences in the maturation kinetics, clathrin content, and actin dependency of clathrin endocytic structures internalizing VSV or DI-T virions. Our principal finding is that, unlike VSV uptake, DI-T internalization does not induce or require robust actin polymerization. We have also established, for the first time, that the geometry of an endocytic cargo can alter the mechanism of clathrin uptake. We propose that VSV-containing clathrin structures display characteristics of ‘frustrated’ endocytic intermediates that cells resolve by using the force of actin assembly to deform the plasma membrane into a complete endocytic vesicle.

## Introduction

Eukaryotic cells internalize constituents of the plasma membrane and extracellular cargos by entrapping them in membrane-bound carriers. The most prominent and well-characterized endocytic carriers are clathrin-coated vesicles (reviewed in [1]–[3]). Coated vesicles transport lipids, proteins, and other essential macromolecules from the cell surface to endosomal organelles. Extensive biochemical and cell biological research supports the following model for conventional coated vesicle formation in higher eukaryotes. The AP-2 clathrin adaptor complex recruits clathrin to the cytosolic leaflet of the plasma membrane and sequesters cargos at the endocytic site [4], [5]. The continued assembly of clathrin into a lattice-like configuration helps deform the underlying membrane and ultimately creates an invagination, or ‘pit’ [2]. Recruitment of the GTPase, dynamin, then facilitates scission of the coated pit from the plasma membrane [6], and clathrin is rapidly removed from the cargo-loaded vesicle by the combined action of the heat shock cognate protein 70 (Hsc70) and its co-chaperone auxilin [7], [8]. The entire process is typically complete within 30–60 s [9], [10].

Coated pits incorporate and internalize soluble cargos of various sizes, such as transferrin (5 nm) [9], [11] and low density lipoproteins (25 nm) [9], [12]. Many viruses and intracellular bacteria are also internalized by the clathrin machinery [9], [13]–[16]. We previously evaluated how cells internalize the 70×200 nm bullet-shaped vesicular stomatitis virus (VSV). We found that VSV internalization occurs through elongated, partially clathrin-coated structures that have longer lifetimes (∼2 min.) than typical endocytic clathrin-coated vesicles and require local actin polymerization for uptake [15]. During VSV internalization, the clathrin coat first assembles as a partially closed dome at one end of the virion [15], [17], and growth of the coat stalls when it encounters the long particle axis. Actin assembly then drives one or more late stage(s) of the internalization process, as recruitment of the actin machinery peaks during completion of clathrin assembly, and pharmacological inhibition of actin polymerization blocks VSV internalization without interfering with clathrin coat assembly [15]. Relatively small, spherical viruses like dengue virus (50 nm) [13] and some influenza A viruses (X-31 strain, ∼120 nm) [14], [18] also enter using a clathrin-dependent route, but it is unclear whether actin function is required for their uptake. Our observations with VSV led us to hypothesize that the physical dimensions of the virion block the ongoing polymerization of clathrin during its uptake, and that the stalled structure recruits regulators of actin assembly whose activity is required to complete the internalization process.

Defective interfering (DI) particles arise spontaneously during virus replication. Such particles depend upon coinfecting helper virus to support their replication but contain all the essential *cis-*acting regulatory elements for genome replication and assembly. One such well- characterized DI particle of VSV is termed DI-T, which lacks 82% of the viral genome [19], [20]. Since the length of a VSV particle is dictated by the genome size [21], DI-T particles are 75 nm long and appear as truncated bullets by electron microscopy [22]. DI-T particles contain normal proportions of the viral structural proteins [23], including the viral surface glycoprotein (G), which mediates VSV attachment and entry into host cells.

Here we took advantage of significant differences in the physical dimensions of VSV and DI-T to investigate how the geometry of a viral cargo influences the actin-dependency of clathrin internalization. Using live cell fluorescence microscopic imaging, we compared the uptake mechanisms of VSV and DI-T at the single particle level. We report that in contrast to the clathrin- and actin-dependent uptake of VSV, the shorter DI-T particles enter cells through fully coated clathrin carriers that do not require actin dynamics for vesicle budding. These observations highlight the plasticity of the clathrin endocytic system, where clathrin coats serve as a scaffold to direct actin assembly when the clathrin machinery alone is not sufficient to mediate internalization.

## Results

### Biological properties of DI-T particles

**Figure 1:**
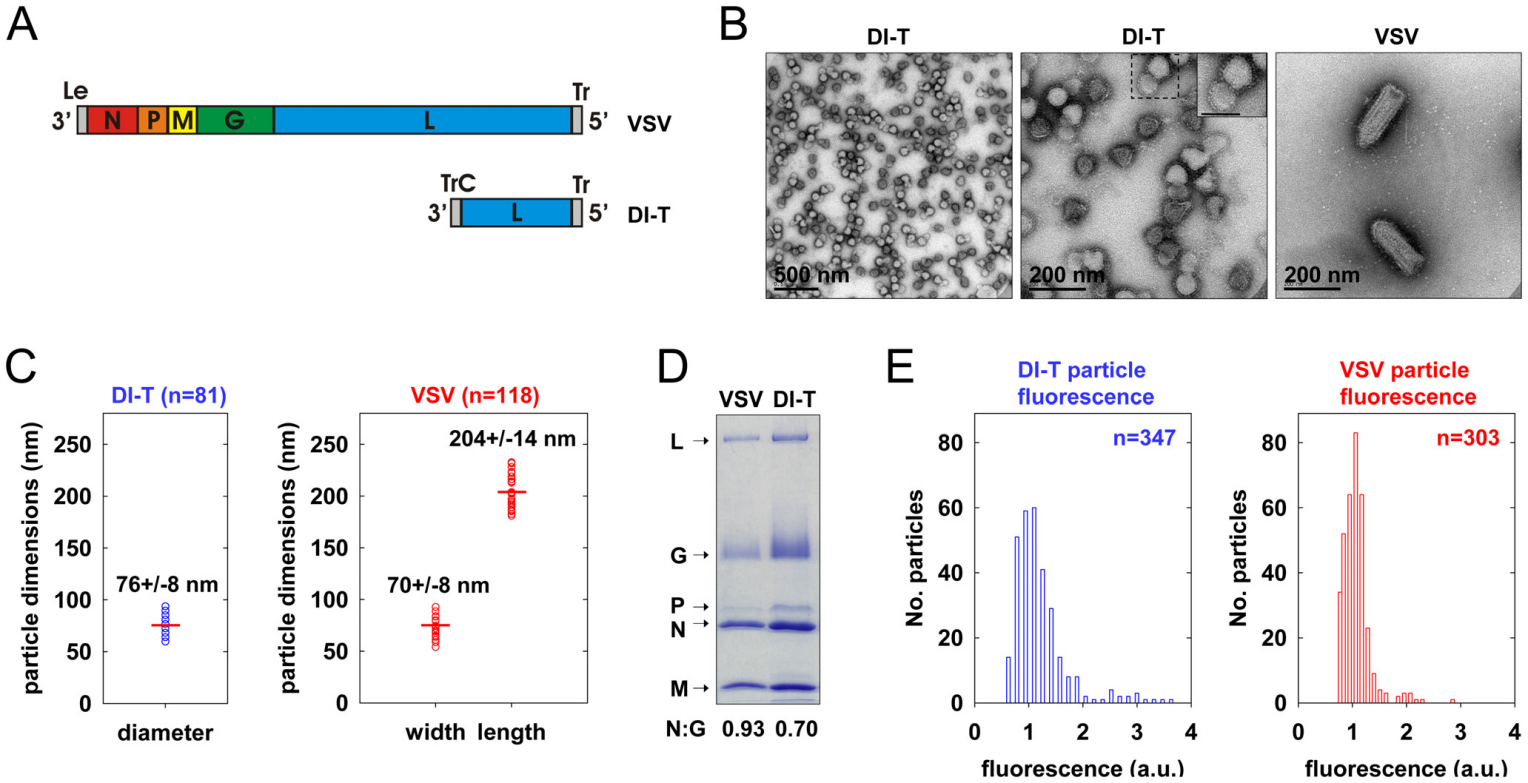
Biological properties of DI-T particles. (A) Structure of VSV and DI-T genomic RNAs. The single-stranded negative-sense RNA genomes are shown in a 3′-5′ orientation. The five viral genes are colored as follows: red nucleocapsid (N) protein gene; orange, phosphoprotein (P) gene; yellow, matrix (M) protein gene; green, glycoprotein (G) gene; blue, large (L) polymerase protein gene. The noncoding genomic terminal leader (Le) and trailer (Tr) regions, which serve as promoters for RNA synthesis and genomic RNA encapsidation, are shown in gray. The DI-T genome comprises 2,208 nts. The 5′ terminal 2,163 nts derive from the 5′ terminus of the parental VSV genome (11,161 nts), and the 3′terminus contains a 45 nt inverted complement of the wild-type Tr (TrC). (B) Electron micrographs of purified VSV and DI-T particles negatively stained with PTA. Middle panel, inset shows an expanded view of virions from the boxed region to facilitate visualization of the viral glycoprotein spikes. Inset scale bar, 100 nm. (C) Virion geometry. The dimensions of individual DI-T (blue) and VSV (red) particles measured from electron micrographs of negatively stained particles. Each open circle represents the measurement for a single particle. Horizontal red lines denote the mean value of each population, and the numerical means (+/− SD) are provided above each plot. (D) Protein composition of purified VSV and DI-T particles. Viral proteins (L, G, P, N, and M) were separated by SDS-PAGE and visualized with Coomassie blue staining. The ratio of N protein to G protein was quantified using ImageJ and is displayed below the gel as a comparative measure of average virion surface glycoprotein density in each particle population. (E) Fluorescence intensity of virus particles labeled with Alexa Fluor dye molecules. Purified DI-T or VSV particles were labeled with Alexa Fluor 647 or 568 and imaged on separate glass coverslips using a spinning disk confocal microscope. The fluorescence intensity of individual spots in a single field of view was quantified, and the distribution of intensity values (in arbitrary units, a.u.) for DI-T (blue) and VSV (red) particles is shown.

To generate a clonal population of VSV DI-T particles, we recovered DI-T from cDNA (Figure 1A) [24] and amplified the particles by co-infection of cells with VSV [25]. We separated DI-T particles from VSV by rate zonal centrifugation in a sucrose density gradient. Electron microscopic analysis (Figure 1B, C) confirmed that VSV virions measure 70+/−8 nm by 204+/−14 nm (n = 114) [21], while the shorter DI-T particles have a length of 76+/−8 nm (n = 81) [22]. DI-T particles, like VSV, are covered with spike-like projections that correspond to homotrimers of G protein (Figure 1B), and SDS-PAGE analysis of purified particles confirmed that VSV and DI-T particles contain similar ratios of G protein to core virion components (Figure 1D) [23]. The purified stocks of DI-T lacked full-length virions (Figure 1B), with only a single VSV virion observed amongst more than 3,000 DI-T particles. Limited dilutions of the purified DI-T stock contained ∼1x10^6^ plaque forming units of virus per microgram of total viral protein, or 10^-5^ times fewer infectious particles than for an equivalent protein quantity of VSV particles (not shown). Thus, we have successfully purified relatively homogeneous populations of VSV and DI-T particles, which differ only in their physical dimensions.

### DI-T particles enter cells by clathrin-dependent endocytosis

**Figure 2:**
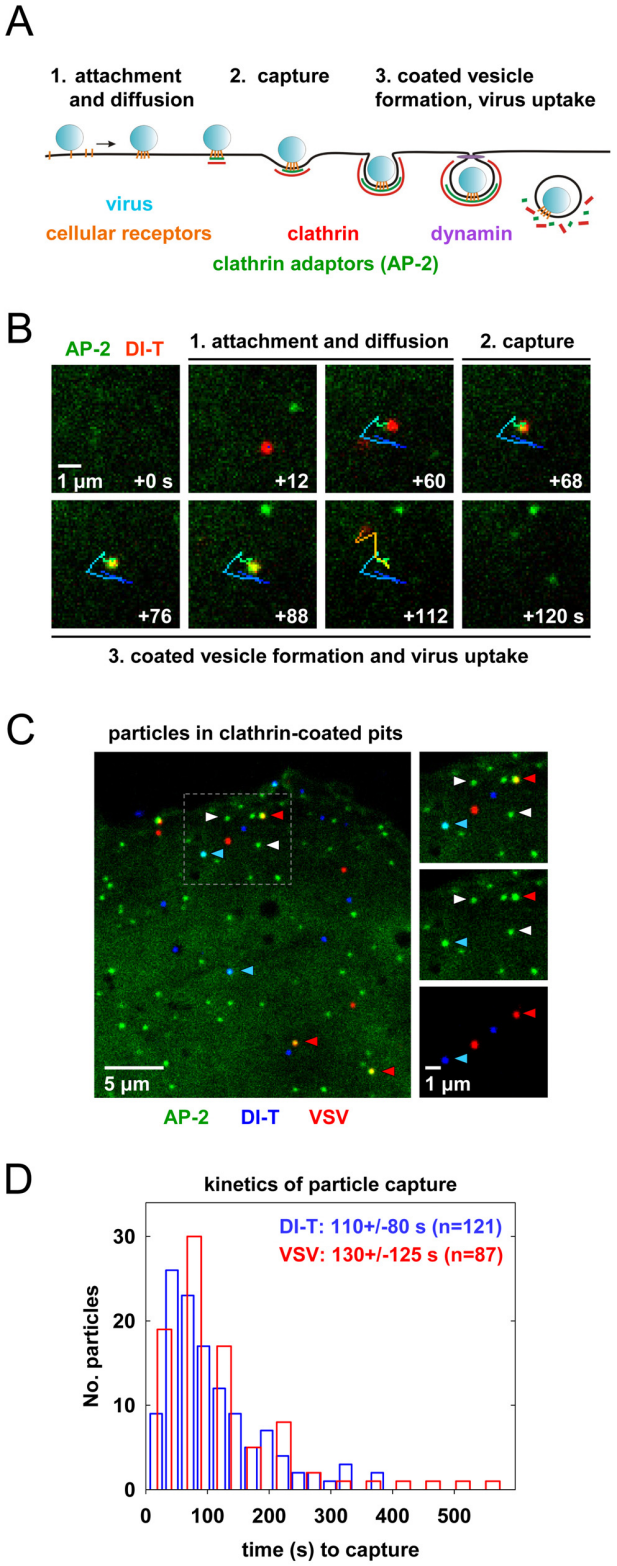
Clathrin structures capture VSV and DI-T particles with similar kinetics. (A) Schematic of clathrin-dependent virus internalization. 1. A particle (blue) attaches to receptor moieties (orange) on the cell surface (black horizontal line), and the virus-receptor complex diffuses in the plane of the membrane. 2. The virus particle is captured by the clathrin endocytic machinery (AP-2, green; clathrin, red) after diffusion into an existing clathrin structure (e.g. Dengue virus) or entrapment within a clathrin structure that initiates in close proximity to the virion (e.g. VSV and influenza A virus). 3. Clathrin assembly completes, and the virus-containing pit is severed from the cell surface in a dynamin-dependent process. Internalization of VSV also requires local actin assembly. Clathrin is rapidly removed from the nascent vesicle, and the vesicle is actively transported further into cell. (B) Example of a complete DI-T internalization event. A single DI-T particle (red) attaches to a BSC1 cell expressing σ2-eGFP (green) and diffuses on the cell surface. A dim spot of AP-2 appears beneath the virion, signifying capture of the particle. The AP-2 fluorescence intensity increases as the clathrin coat assembles, and the virus disappears into the cell shortly after the AP-2 signal reaches a maximum (Video S1). Numbered stages correspond to the events described in A. The path of particle motion is depicted as a linear, color-coded trace that progresses with time from blue to red. (C) VSV and DI-T particle capture by clathrin structures in the same cell. BSC1 cells stably expressing σ2-eGFP (green) were inoculated with Alexa Fluor 647-labeled DI-T (blue, blue arrowheads) and Alexa Fluor 568-labeled VSV (red, red arrowheads). Time-lapse images were acquired at 4 s intervals using a spinning disk confocal microscope. Left, snapshot of a cell depicting coated pits lacking (white arrowheads) or containing (blue/red arrowheads) virus particles. Right, expanded split-channel views of the region within the dashed box at left. (D) Kinetics of virus capture. BSC1 cells stably expressing σ2-eGFP (7 cells) or eGFP-LCa (12 cells) were inoculated with VSV and DI-T particles. Images were acquired at 3-4 s intervals as in C, and the time interval between virus attachment and detection of AP-2 or LCa beneath a virion was quantified for productive internalization events. The distribution of capture times is shown for DI-T (blue) and VSV (red) particles, and the mean time to capture (+/− SD) for each particle population is provided at right. The kinetics of VSV and DI-T capture are not significantly different (Student's *t*-test p value = 0.2).

To visualize VSV and DI-T by fluorescence microscopy, we covalently labeled the G proteins with spectrally separable fluorescent dye molecules (Alexa Fluor 568 and 647, respectively) using conditions that do not reduce viral infectivity [15]. Spinning disk confocal images of labeled particles adsorbed onto glass coverslips showed diffraction-limited objects with single-peaked distributions of fluorescence intensity values (Figure 1E), indicating that the DI-T and VSV populations primarily consist of individual particles [15]. We tracked the entry of DI-T particles into BSC1 cells stably expressing an eGFP-tagged σ2 subunit of the AP-2 adaptor complex (σ2-eGFP), which incorporates into all clathrin-coated structures that form on the plasma membrane [9], [15], [26]. Single DI-T particles readily attached to cells and progressed through the following set of defined events (see Figures 2A, B; Video S1 for examples): (1) membrane-bound DI-T particles diffused slowly (D  = 5×10^−11^–5×10^−12^ cm^2^ s^−1^) and with the random directionality characteristic of Brownian motion; (2) shortly after DI-T attachment, a dim spot of AP-2 signal arose and remained colocalized with the particle, signifying incorporation of DI-T into an assembling clathrin-coated pit; (3) the AP-2 signal steadily increased over time until it peaked as coat assembly completed, and the DI-T particle then underwent an abrupt movement into the cell, after which the AP-2 signal disappeared due to clathrin uncoating. This sequence of events is identical to what we previously observed for VSV entering cells by clathrin-dependent endocytosis [15]. Moreover, the efficiency of DI-T uptake *via* the clathrin pathway is similar to that of the full-length VSV particles, as 89% (55/62) of DI-T particles that attached to 3 individual cells during imaging entered by clathrin-dependent endocytosis. These data show that DI-T efficiently enters cells through the clathrin pathway and validate the use of DI-T and VSV as comparative endocytic cargos.

### Clathrin structures capture VSV and DI-T particles with similar kinetics

To directly compare how DI-T and VSV particles engage the clathrin machinery, we simultaneously inoculated BSC1 cells with the two spectrally distinct particle forms and then analyzed their mode of incorporation into AP-2 containing clathrin structures on a single cell basis (Figure 2C). For each complete virus uptake event, we quantified the kinetics of particle capture by measuring the elapsed time between virion attachment and the appearance of an AP-2 signal that colocalized with the bound particle. The capture time for DI-T particles was 110+/−80 s (n = 121), which is statistically indistinguishable (Student's *t-*test, p = 0.2) to that measured for VSV (130+/−125 s (n = 87)) and agrees well with our prior measurements (Figure 2D) [15]. The AP-2 structures that captured DI-T or VSV initiated within a ∼250 nm zone (the resolution limit of the optical system) of the attached particle. We therefore conclude that DI-T and VSV particles engage the clathrin system in an indistinguishable manner, which likely reflects a shared mechanism triggered by the same viral glycoprotein-receptor interactions.

### Cells internalize DI-T particles using conventional clathrin-coated vesicles

**Figure 3:**
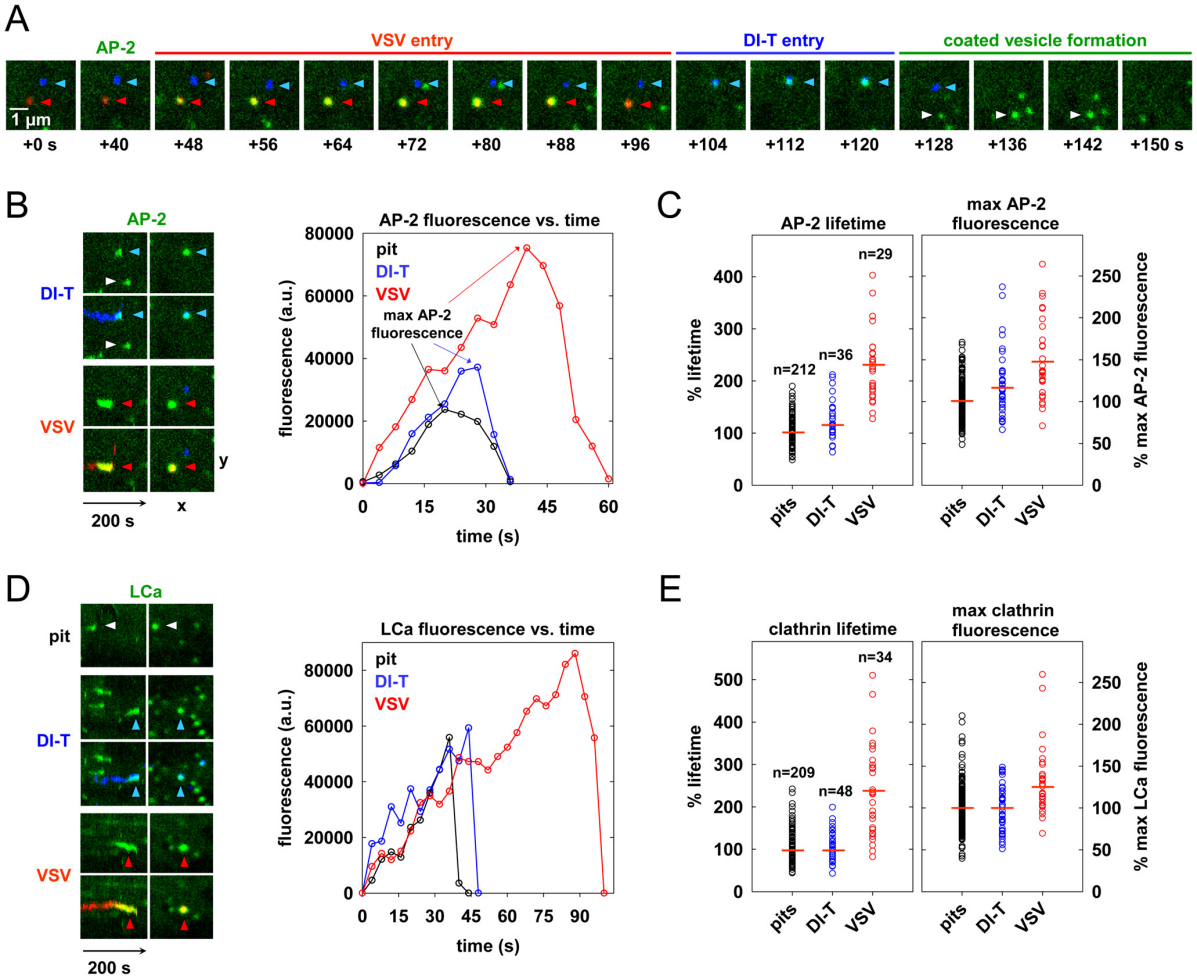
Cells internalize DI-T particles using conventional clathrin-coated vesicles. (A) Internalization of VSV and DI-T by the same cell. BSC1 cells stably expressing σ2-eGFP (green) were inoculated with VSV (red) and DI-T (blue) particles, and confocal images were captured at 4 s intervals. The images show the sequential internalization of single VSV (red, red arrowheads) and DI-T (blue, blue arrowheads) particles, followed by the formation of a canonical coated vesicle (white arrowhead) within a 3.5×3.5 µm^2^ area of the plasma membrane (Video S2). The first acquired frame of the time-lapse series is designated +0 s, and the capture time of the subsequent images is shown. (B) AP-2 recruitment during the uptake events shown in A. Left, image quadrants depicting AP-2 accumulation over time (left panels) and at the time of maximum AP-2 signal during each event (right panels). Upper panels in each quadrant show the AP-2 channel alone, and the lower panels show overlays of the virus and AP-2 channels. The highlighted pit from A. is indicated with a white arrowhead, and virus particles are colored as in A. Right, fluorescence intensity (in arbitrary units, a.u.) of AP-2 over time for the events shown at left and in A. The time of AP-2 detection above the local background was set to t = 0 s for each event. (C) Kinetics and AP-2 content of endocytic structures. The plots show the relative lifetime (left) and maximum fluorescence intensity (right) of AP-2 during the uptake of coated pits lacking virus (pits, black) or structures that internalized DI-T (blue) or VSV (red) particles (from 4 cells). Values are expressed as percentages to facilitate comparison of viral and nonviral uptake events across multiple cells. Approximately 50 pits lacking virus were analyzed in each cell, and the mean of the measured values was calculated for each parameter. The values for each nonviral and viral uptake event were divided by the mean for pits lacking virus in the same cell, and the resulting values were multiplied by 100. Each open circle represents a single uptake event, and horizontal red lines demark the mean of the compiled population. The number of events is provided above each plot. Numerical values and statistical analyses are provided in Table 1. (D) Clathrin recruitment during virus entry. Left, kymograph views of internalization events from a single BSC1 cell transiently expressing eGFP-LCa (Videos S3, S4). Images were captured as in A. and displayed as described in B. Right, fluorescence intensity of eGFP-LCa over time for the events shown at left. (E) Kinetics and clathrin content of endocytic structures. The plots show the relative lifetime (left) and maximum fluorescence intensity (right) of clathrin during the uptake of coated pits lacking virus (pits, black) or structures that internalized DI-T (blue) or VSV (red) particles (from 3 cells). Data were calculated and plotted as described in C. Numerical values and statistical analyses are provided in Table 1.

To investigate the characteristics of the clathrin coat responsible for DI-T internalization, we imaged the uptake of both particle forms by the same BSC1 cell. We found that DI-T particles are internalized through AP-2 containing structures significantly faster than full-length virions (Figure 3A, B; Video S2). Quantitative analysis of data compiled from 4 cells showed that pits incorporating DI-T (n = 36) form in 43+/−14 s, which is similar to the assembly kinetics of pits that lack virus particles (n = 212, 35+/−10 s) (Figure 3C, Table 1). As expected, AP-2 structures that capture VSV (n = 29) require longer (75+/−22 s) to complete (Figure 3C, Table 1). A similar analysis conducted in cells transiently expressing eGFP-tagged clathrin light chain A1 (eGFP-LCa) yielded analogous results (Figure 3D, E, Table 1, Videos S3, S4). The interaction of full-length VSV with a cell had no impact on the uptake kinetics of DI-T into the same cell (Table 1).

**Table 1:** Summary of kinetic and fluorescence intensity data.

Particle(s)	Fluorescent protein(s)	Treatment	# Expts/# Cells	Events analyzed	# Events	Lifetime	% Lifetime	% Max fluorescence
DI-T	σ2		1/3	DI-T	62	47+/−13 s	ND	ND
DI-T+VSV	σ2		1/4	pits	212	35+/−10 s	100+/−24	100+/−23
				DI-T	36	43+/−14 s	128+/−37**	119+/−38**
				VSV	29	75+/−22 s	224+/−68**	148+/−48**
	LCa		1/3	pits	209	44+/−19 s	100+/−34	100+/−26
				DI-T	48	44+/−14 s	100+/−30	99+/−26
				VSV	34	89+/−36 s	222+/−104**	130+/−38**
DI-T+VSV	σ2	+latB	2/4	pits	193	47+/−14 s	100+/−27	100+/−27
		+latB		DI-T	46	54+/−21 s	118+/−46*	133+/−42**
		+latB		VSV entry	20	141+/−104 s	298+/−198**	207+/−59**
		+latB		trapped VSV	31	615+/−216 s	1283+/−430**	192+/−73**
DI-T	LCa + cortactin		1/3	pits	155	51+/−19 s	100+/−34	100+/−50
				DI-T	30	65+/−28 s	129+/−63*	130+/−63*
			1/3	pits	220	52+/−24 s	100+/−36	100+/−66
VSV	LCa + cortactin			VSV	21	166+/−63 s	335+/−105**	300+/−164**

Kinetic and fluorescence values are provided as the mean +/− SD for all events in a given context. The number of experiments and cells analyzed is indicated. The absolute lifetimes are expressed in seconds (s). The % lifetime and % maximum fluorescence intensity values were calculated as described in the legend of Figure 3C. The % maximum fluorescence intensity of cortactin is provided for events analyzed in cells co-expressing mCherry-LCa and cortactin-eGFP. A two-tailed Student's *t*-test was used to determine whether data from 2 categories differ in a statistically significantly manner.

(*) denotes a statistical difference with a p-value <0.005 and >0.00005 when comparing data in a given category to data for pits lacking virus in the same category.

(**) similarly denotes a p-value <0.00005. All values for VSV are significantly different (p<0.005 for % max AP-2 fluorescence; p<0.00005 for all other categories) from those measured for DI-T particles in the same context. ND, not determined.

**Figure 4:**
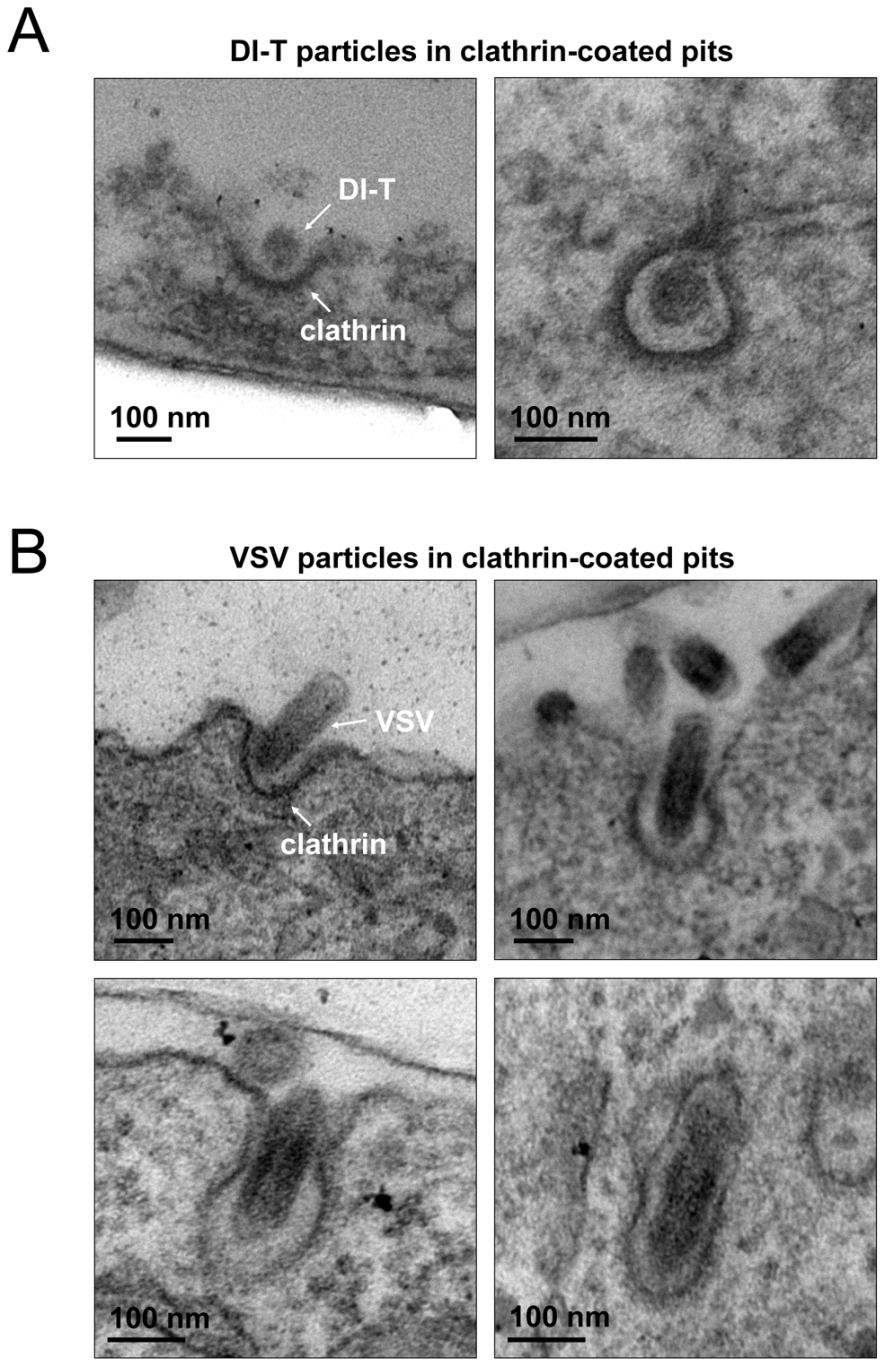
Electron microscopic images of DI-T and VSV particles in clathrin-coated pits. (A) Electron micrographs depicting DI-T particles at early (left) or late (right) stages of clathrin-dependent endocytosis. BSC1 cells were incubated with ∼1000 particles per cell for 10 min. at 37°C. Cells were then fixed, and samples were processed for ultra thin sectioning and viewed as described in the materials and methods. (B) Electron micrographs of VSV particles at sequential (left to right, top to bottom) stages of clathrin-dependent endocytosis. Vero cells were inoculated with VSV at an MOI of 5, and samples were prepared for analysis at 6 h post-infection.

The different kinetics of DI-T versus VSV internalization suggests that DI-T enters cells through conventional fully coated clathrin structures and not the partially coated vesicles responsible for VSV uptake. To further investigate this possibility, we measured the maximum fluorescent signal of eGFP-tagged AP-2 or clathrin molecules, as this peak signal is known to be proportional to the overall size of a clathrin coat [9], [15], [26]. We compared this value for structures that contained DI-T with those that contained VSV or lacked either particle. Similar quantities of coat components were present in structures associated with DI-T to those lacking viral particles (Figure 3C, E, Table 1). As expected, VSV-containing structures accumulate more AP-2 and LCa molecules than structures lacking virus (Figure 3C, E, Table 1). Taken together, the above experiments suggest that DI-T enters cells through pits that acquire a full clathrin coat. Consistent with this, electron micrographs of DI-T particles captured during cell entry show particles present in circular pits entirely surrounded by a clathrin coat (Figure 4A). This is in marked contrast to the partial clathrin coat found at one end of the endocytic carriers that internalize VSV (Figure 4B) [15].

### Clathrin structures containing VSV recruit more cortactin that pits that internalize DI-T

**Figure 5:**
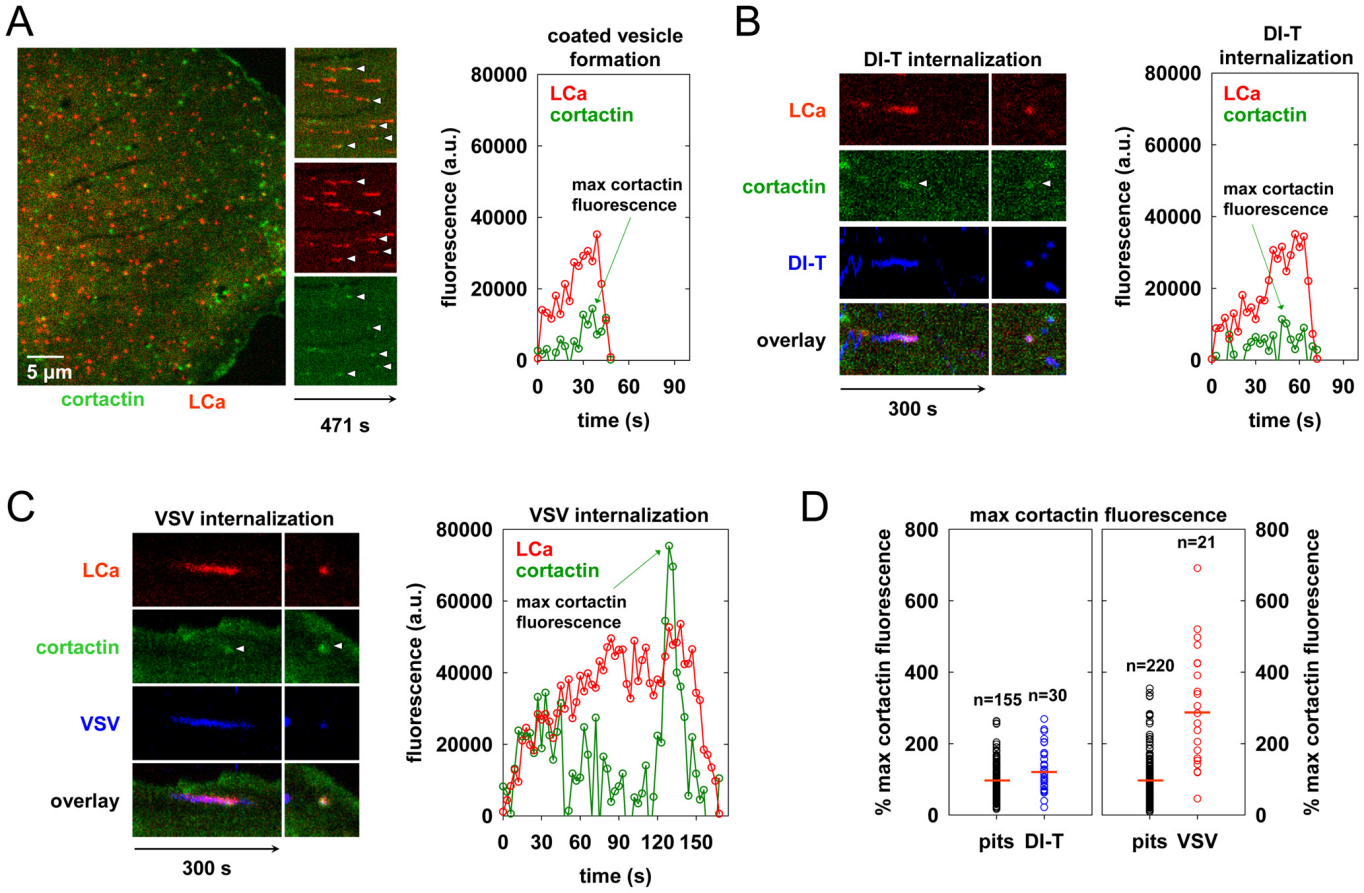
Clathrin structures containing VSV recruit more cortactin that pits that internalize DI-T. (A) Cortactin recruitment during coated pit formation. Left, snapshot showing the surface of a BSC1 cell transiently expressing cortactin-eGFP (green) and mCherry-LCa (red) at 18 h post-transfection. Time-lapse images were acquired at 3 s intervals, and frame 83 is shown. Middle, split channel kymographs of coated pit formation in the cell at left. White arrowheads highlight pits in which cortactin recruitment is clearly visible above the local background. Right, example plot of cortactin and clathrin fluorescence intensity over time during the formation of a single clathrin-coated pit in the cell shown at left (Video S5). (B and C) Examples of cortactin recruitment during DI-T (B) and VSV (C) uptake. BSC1 cells transiently expressing mCherry-LCa (red) and cortactin-eGFP (green) were inoculated with Alexa Fluor 647-labeled DI-T or VSV, and images were acquired as in A. Left, split-channel kymograph views of protein and virion (blue) fluorescence intensity over time (Videos S6, S7). Images in the right-hand panels show a snapshot of the maximal cortactin or clathrin fluorescence, and white arrowheads highlight the peak cortactin signal. Right, plots of the cortactin and clathrin fluorescence intensity over time for each internalization event. (D) Relative peak fluorescence intensity of cortactin in cells co-expressing mCherry-LCa and cortactin-eGFP. At 18 h post-transfection, samples were separately inoculated with DI-T or VSV particles, and images were acquired as in A. For each cell that was imaged, the maximum cortactin fluorescence associated with ∼50 pits lacking virus particles (pits, black) and all pits that internalized a DI-T (left, blue, 3 cells) or VSV (right, red, 5 cells) particle was measured. The data are plotted as described in the legend of Figure 3C, and the number of events is shown above each plot. Numerical values and statistical analyses are provided in Table 1.

During the final phase of coat assembly, endocytic clathrin structures associated with VSV show a strong recruitment of cortactin, an F-actin and dynamin binding protein that activates Arp2/3-mediated assembly of branched actin filaments [15], [27], [28]. To determine whether DI-T uptake is associated with an acute recruitment of cortactin, we monitored internalization into BSC1 cells transiently co-expressing monomeric Cherry-LCa (mCherry-LCa) and low levels of cortactin-eGFP. As previously shown [15], [26], [29], conventional clathrin-coated pits exhibit minimal cortactin recruitment that typically peaks just before completion of clathrin assembly (Figure 5A, Video S5). Cortactin recruitment is similarly sparse during the uptake of DI-T particles (Figure 5B, Video S6). In marked contrast, and as expected [15], large bursts of cortactin accompany the internalization of VSV (Figure 5C, Video S7). Quantitative analysis revealed that the peak fluorescence intensity of cortactin detected in the late phase of VSV uptake averaged 3-fold higher than the signal associated with pits containing DI-T or pits that did not capture a virus particle (Figure 5D, Table 1). These data suggest that while formation of short-branched actin filaments is required during the late stages of clathrin-mediated VSV entry, this need is obviated during clathrin-dependent DI-T entry.

### Actin polymerization is not required for DI-T internalization

**Figure 6:**
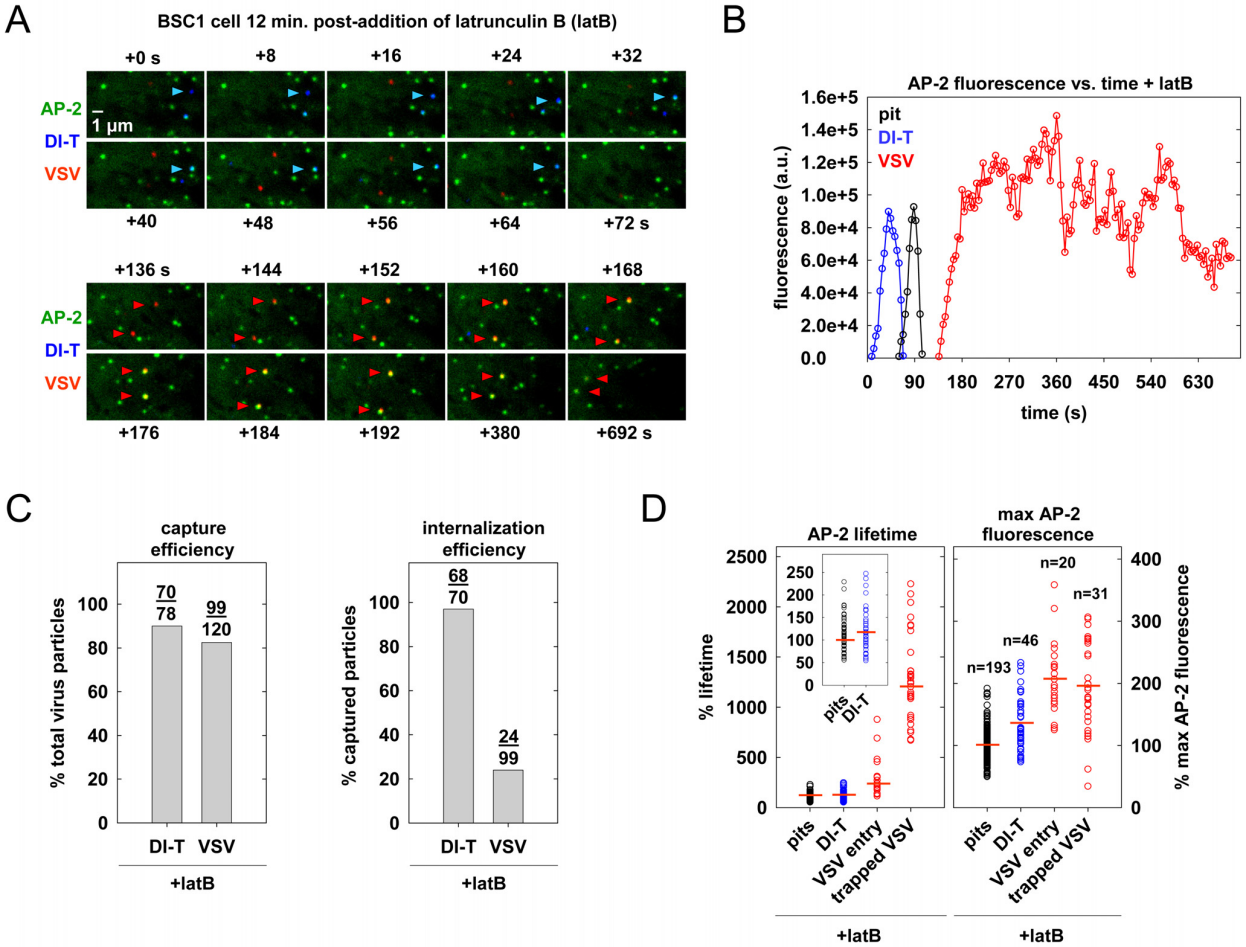
Actin polymerization is not required for DI-T internalization. (A) The endocytic fate of virus particles after inhibition of actin polymerization. BSC1 cells stably expressing σ2-eGFP (green) were treated with 6.3 µM latB for 12 min. and inoculated with DI-T and VSV particles in the continued presence of latB. Time-lapse images of a single cell were acquired at 4 s intervals for 692 s, and an 8.8×5.0 µm^2^ area of the cell surface is shown. The upper panels show the complete internalization of a DI-T particle (blue, blue arrowheads), where +0 s indicates the first frame of the time-lapse series. The lower panels show the subsequent capture but failed internalization of 2 VSV (red, red arrowheads) particles on the same area of cell membrane (time scale continued from above) (Video S8). (B) AP-2 fluorescence intensity for the events shown in A. Note that the adaptor fluorescence associated with the DI-T particle (blue) and a conventional coated pit (black) that formed within the same membrane area peak and disappear normally, while the adaptor signal associated with the upper-most VSV particle (red) does not, signifying failed internalization. (C) Effect of latB on the efficiency of virus capture and internalization. BSC1 cells stably expressing σ2-eGFP were treated and imaged as described in A. Left, the percentage of virus particles that were captured by a clathrin structure after attachment. Right, the percentage of captured virus particles that were successfully internalized within 300 s of capture (see D. for details). Cumulative data are from 5 cells. (D) Effect of latB on the lifetime and peak fluorescence intensity of AP-2 in clathrin structures. Data were acquired as described in A. and displayed as in the legend of Figure 3C. Left, relative lifetime of AP-2 in structures that lack (pits, black) or capture a virus particle. Inset shows a rescaled distribution of the pit and DI-T internalization events. Right, maximum fluorescence intensity of AP-2 in the events at left. Data are from 4 of the 5 cells analyzed in C, as thermal drift during imaging prevented accurate fluorescence intensity measurements in one cell. The number of events in each category is shown above the corresponding plots at right. DI-T (blue) data consists only of productive internalization events. VSV events are categorized as productive internalizations (VSV entry, red) or non-productive captures (trapped VSV, red). A non-productive capture is defined as a stable colocalization between a spot of AP-2 and a VSV particle that began at least 300 s before the last captured image and did not result in virus uptake before cessation of imaging. The 300 s cutoff was chosen because a majority (22/24) of productive internalizations occurred within 300 s of capture. Captures in which the final image frame was acquired before 300 s elapsed were excluded from the analysis, as the eventual endocytic fate of the particle cannot be predicted.

To directly test whether actin assembly is required for DI-T entry, we treated BSC1 cells with latrunculin B (latB), a chemical inhibitor of actin filament assembly [30], and tracked the endocytic fate of DI-T and VSV in the same cells. Treatment of cells with 6.3 µM latB did not change the efficiency of DI-T entry (Figure 6A–C, Video S8), but it reduced the internalization of VSV by >75% (Figure 6A–C). As expected [15], latB treatment did not affect the capture efficiency of either particle type by clathrin (Figure 6C). The lifetimes and AP-2 content of pits lacking particles or containing DI-T was similarly unaffected by latB (Figure 6D). We conclude that the shorter DI-T particles bypass the actin requirement displayed by the larger VSV for efficient clathrin-based uptake.

## Discussion

The major conclusion of our study is that the physical properties of a virus particle dictate the need for engagement of the actin system during its clathrin-dependent uptake into cells. We formulate this conclusion based on tracking the clathrin-dependent internalization of VSV and its shorter DI-T particle into live cells. Internalization of VSV is accompanied by the recruitment of cortactin at a late step of the endocytic process, and chemical inhibition of actin polymerization blocks virus uptake (this study and [15]). By contrast, internalization of the shorter DI-T particles is insensitive to chemical inhibition of actin polymerization and is not accompanied by the same spike of cortactin recruitment observed for the full-length VSV particles. VSV and DI-T particles differ in their length and not the density of the glycoprotein spike that dictates their entry. We suggest that the shape of the full-length VSV particles presents a physical barrier that frustrates completion of the clathrin-coated pit. The stalled clathrin structure then recruits the actin machinery required to finalize internalization. The shorter DI-T particles no longer provide such a physical barrier during engulfment, which results in their actin-independent internalization through coated vesicles that acquire a full complement of clathrin.

### The importance of particle length for the clathrin-dependent uptake of VSV

Receptor-dependent signaling events lead to actin filament assembly during the clathrin-independent uptake of other viruses, including poliovirus and coxsackie B virus [31], [32]. Here we show that the initial interactions of DI-T with the cell are indistinguishable to those of VSV, as both particle types diffuse slowly on the cell surface and are captured by coated pits with similar kinetics (Figure 2). Such similar behavior suggests that DI-T and VSV engage an as yet unknown viral receptor in an analogous manner. Since initial receptor interactions appear indistinguishable, it seems remote that VSV G-receptor interactions induce a signaling cascade that leads to actin polymerization at sites of VSV but not DI-T uptake. We therefore conclude that the glycoprotein is not the primary trigger of actin assembly during clathrin-dependent VSV internalization.

**Figure 7:**
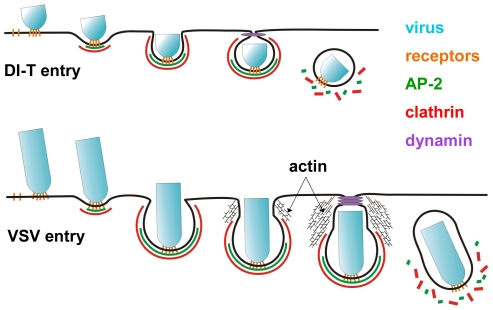
Model of DI-T and VSV entry. DI-T (above) and VSV (below) particles engage host cells through interactions between the viral surface glycoproteins and unknown cellular receptor moieties. Following attachment, both particle types undergo slow diffusion (diffusion coefficient ∼5×10^−11^ cm^2^ s^−1^) on the cell surface for an average of ∼2 min. before being captured by a clathrin-coated pit. For DI-T, continued clathrin assembly drives complete particle envelopment by the plasma membrane and leads to virus endocytosis. In contrast, the presence of a VSV particle in a coated pit physically prevents complete membrane constriction by the clathrin machinery and causes clathrin assembly to halt prematurely. The force provided by actin polymerization then further remodels the plasma membrane and thereby encloses the virus particle in a partially clathrin-coated vesicle.

We previously showed that endocytic structures containing VSV do not acquire a full clathrin coat [15], which agreed with earlier electron micrographs depicting virus particles in tubular invaginations with clathrin at the cytosolic tip [17]. The morphology of those structures suggests that cells initially capture one tip of the virus particle, and clathrin assembly stalls when the constricting coat encounters the long axis of the virion (Figure 7). Here we found that DI-T particles do not alter the process of clathrin-coated vesicle formation (Figures 3, 4). This finding implies that clathrin can fully enclose cargos displaying VSV G provided that the particle shape does not physically prohibit clathrin assembly or closure of the plasma membrane (Figure 7). Consequently, the physical properties of the VSV particle captured by a clathrin-coated pit are what dictate the altered mode of actin-dependent uptake. We therefore propose the model (Figure 7) that it is the incomplete clathrin structure formed during VSV uptake that elicits the actin-based response to rescue the endocytic process and leads to the successful engulfment of the trapped virus particle.

### Actin-dependent clathrin-mediated endocytosis

The importance of actin function during clathrin-mediated endocytosis varies. In yeast cells, actin polymerization drives invagination and endocytosis of long-lived (>2 min.) clathrin assemblies through tubular intermediates with clathrin at the cytosolic tip [33]–[35]. Mammalian cells also form large (0.2–1 µm), relatively flat arrays of clathrin, or ‘plaques,’ on the adherent cell surface [26], [36]. These structures also exhibit long lifetimes (2-15+ min.) and require local actin assembly for internalization [26]. Coated pit internalization from the apical (but not the basolateral) surface of polarized mammalian cells [37], [38] and lamprey neuronal synapses [39], [40] is also actin-dependent. By contrast, actin polymerization is only required for the uptake of clathrin plaques and not conventional coated pits in several types of nonpolarized mammalian cells [26], [41]. Thus, while actin dynamics play an evolutionarily conserved role in clathrin-dependent endocytosis, mammalian cells regulate the interplay between the clathrin and actin systems.

Our analyses of VSV and coated plaque internalization reveal two properties that correlate with their actin-dependent uptake mechanisms. First, plaques and VSV-containing structures remain on the plasma membrane for >2-fold longer than standard coated pits [15], [26] (Figure 3C, E). The prolonged presence of a clathrin lattice might promote interactions between clathrin-associated proteins and regulators of actin polymerization. Second, clathrin plaques and VSV-containing structures physically differ from standard coated pits. Plaques fail to constrict their outer boundaries during the final phase of clathrin assembly [26], and pits containing VSV lack a complete clathrin coat [15], [17]. Such unusual structural features might attract proteins that associate with exposed lipids, such as dynamin. Indeed, significantly more dynamin molecules accumulate during the final stages of VSV uptake [15]. This localized increase in dynamin may enhance the recruitment of dynamin-interacting proteins with the capacity to bind lipids and activate the Arp2/3 complex through N-WASP, including endophilin, syndapin, and SNX9 [42]–[46]. Although these proteins may link the clathrin and actin systems and facilitate localized membrane remodeling during endocytosis (reviewed in [47]), further studies are needed to determine whether they play a role in clathrin-dependent VSV endocytosis. Comparative studies of VSV and DI-T uptake may provide a useful tool to dissect the mechanisms that regulate actin assembly during clathrin-mediated endocytosis.

### Implications for the internalization mechanisms of other viruses

The dimensions of the actin-dependent VSV particle and the actin-independent DI-T particle fall within the range of shapes present in many pleomorphic viruses. Our work suggests that this may lead to important distinctions in their mode of uptake. For example, some influenza A virus strains, such as X-31, produce spherical particles that measure 80–120 nm in diameter [18]. By contrast, the Udorn strain forms filamentous particles that measure 80–100 nm wide and up to 30 microns in length [48], [49]. Although influenza A virus can enter cells by clathrin-dependent and -independent mechanisms [14], the impact of particle shape on the entry pathway remains unknown. It seems likely that remodeling of the cortical actin cytoskeleton will be important for uptake of filamentous influenza particles, and clathrin may facilitate local membrane deformation during the endocytic process. The *Arenaviridae* generate roughly spherical particles that range in diameter from 40–300 nm [50], [51], and it is known that clathrin function is important for efficient infection of cells by some New World arenaviruses [52]. It will now be of interest to determine whether spherical particles of different diameter employ altered modes of clathrin-based endocytosis.

Pseudotyping is often used to study the entry pathway of highly pathogenic viruses, including the long filamentous filoviruses, Ebola and Marburg, as well as several arenaviruses. Such pseudotypes are frequently based on VSV or retroviral virions in which the endogenous entry proteins have been replaced with the surface glycoproteins of the pathogenic virus [53]–[56]. Although viral pseudotypes are useful for studying the entry process, VSV and the spherical virions of retroviruses (∼100 nm in diameter) do not accurately recapitulate the sizes or shapes of the pleomorphic viruses. Our studies of VSV and DI-T clearly show that virion geometry can fundamentally alter aspects of the viral internalization process. Therefore, it is critically important to study viral endocytosis using pseudotyped or virus-like particles that closely approximate the physical properties of a virus in question.

## Materials and Methods

### Cells and viruses

African green monkey kidney BS-C-1 cells (herein BSC1, American Type Culture Collection (ATCC) CCL-26; Manassas, VA) and Vero cells (ATCC) were maintained at 37°C and 5% CO_2_ in Dulbecco's Modified Eagle Medium (DMEM, Invitrogen Corporation; Carlsbad, CA) supplemented with 10% fetal bovine serum (Tissue Culture Biologicals; Tulare, CA). BSC1 cells stably expressing rat σ2 adaptin-eGFP (σ2-eGFP) [9] were maintained as above in the presence of 0.4 mg mL^−1^ geneticin (G418, Invitrogen).

Recombinant VSV (rVSV) [25] was amplified and purified as before [15]. Defective interfering T (DI-T) particles of VSV were recovered from a cDNA clone of the DI-T genome [24]. The DI-T particles were amplified by co-infecting baby hamster kidney cells (BHK-21, ATCC C-13) with rVSV (multiplicity of infection (MOI) 50). A subsequent passage was performed by inoculating cells with filtered, undiluted supernatant from the primary amplification and rVSV (MOI of 50). Viruses were concentrated by centrifugation at 44,000× g, and the virus pellet was resuspended in NTE (10 mM Tris pH 7.4, 100 mM NaCl, 1 mM EDTA). The two particle forms were separated on a 15–45% sucrose gradient prepared in NTE by centrifugation at 77,000× g for 5 h. The DI-T particles were extracted from the upper virus band, concentrated as before, and resuspended to 1 mg mL^−1^ of total protein in PBS.

### Dye conjugation to virus particles

Purified DI-T and VSV particles were labeled with Alexa Fluor dye molecules (Molecular Probes, Invitrogen) as previously described [15] except that the final dye concentration in the labeling reaction was reduced to 25 µg ml^−1^. Plaque assays of virus preps before and after labeling showed that dye conjugation did not affect the infectivity of VSV particles or the capacity of DI-T virions to inhibit plaque formation by VSV. The surface density of G protein on VSV or DI-T particles was estimated by measuring the ratio of G protein to N protein in each particle population. To separate and visualize the viral proteins, purified virions were subjected to SDS-PAGE using 10% polyacrylamide and 0.13% bis-acrylamide and stained with Coomassie blue. The relative amounts of N or G protein were established using ImageJ (U. S. National Institutes of Health, Bethesda, Maryland; http://rsb.info.nih.gov/ij/).

### Nucleic acid transfection

BSC1 cells were seeded into 6-well plates at ∼60,000 cells per well 16–20 h prior to transfection. Plasmid DNA was introduced into the cells using FuGENE 6 (Roche Diagnostics; Indianapolis, IN) according to the manufacturer's instructions. Prior to transfection, media on the cells was replaced with 1 ml OPTIMEM (Invitrogen). After addition of the transfection mixture, cells were incubated at 37°C for 5 h, and the existing media was supplemented with 2 ml DMEM containing 10% FBS. To ensure optimal replacement of endogenous clathrin light chain molecules with rat eGFP-clathrin light chain A1 (eGFP-LCa) [9], cells were transfected with 0.75 µg of plasmid DNA encoding eGFP-LCa. The cells were cultured for ∼36 h and seeded onto glass coverslips ∼14 h prior to image acquisition. Co-expression of mCherry-LCa (constructed as described for tomato-LCa [8]) and mouse cortactin-eGFP [57], [58] was achieved by transfection of cells on glass coverslips with 1 µg of each plasmid, and the cells were imaged ∼18 h later.

### Live cell imaging

Cells on 25 mm coverslips (No. 1.5, Electron Microscopy Sciences; Hatfield, PA) were placed into a perfusion chamber and overlaid with α-MEM lacking phenol red (Invitrogen) and supplemented with 20 mM HEPES pH 7.4 and 2% FBS. The chamber was placed in a heated sample holder (20/20 Technology Inc.; Wilmington, NC) mounted on the stage of a Mariana imaging system (Intelligent Imaging Innovations, Denver, CO) based on an Axiovert 200M inverted microscope (Carl Zeiss, Inc.; Thornwood, NY). The microscope stage and objective lenses were maintained at 37°C within an environmental chamber, and the air above the cells was supplied with 5% CO_2_. The position of the sample holder with respect to the objective lens was manipulated using a PZ-2000 automated stage (Applied Scientific Instrumentation; Eugene, OR). Samples were illuminated using 40–50 mW solid state lasers (λ = 488, Coherent, Inc.; Santa Clara, CA, λ = 561, Cobolt AB; Solna, Sweden, λ = 640, 40 mW; Coherent) directed through an acousto-optic tunable filter (AOTF; Gooch and Housego L.L.C.; Melbourne, FL). Laser illumination was imparted on the sample through a CSU-X1 spinning disk confocal unit (Yokogawa Electric Corporation; Tokyo, Japan) and a 63X objective lens (Plan-Apochromat, NA 1.4, Carl Zeiss). Emission spectra were selected using single band width emission filters (LF405/488/561/635-A, Semrock; Rochester, NY), and after transmission through a spherical aberration correction device (Infinity Photo-Optical; Boulder, CO), the emission photons were collected using a Cascade II:512B back-illuminated EMCCD camera (Roper Scientific, Photometrics; Tuscon, AZ). Under this configuration, a single pixel corresponds to 0.07×0.07 µm^2^. Slidebook 4.2.13 (Intelligent Imaging Innovations, Inc. (III); Denver, CO) was used to command the hardware devices and visualize the acquired data.

To image virus internalization, Alexa-labeled virus was centrifuged briefly to remove aggregates, and cells were inoculated with virus to result in attachment of <50 particles per cell within 20 min. (∼1×10^7^ p.f.u. of VSV, ∼MOI 100, 0.005–0.05 particles per µm^2^ of cell surface area). Time-lapse acquisitions were typically carried out for 8–10 min. per cell, and images were captured at 3–4 s intervals after sequentially illuminating the sample with the appropriate laser for 50–100 ms per wavelength. To assess the effect of latrunculin B (latB) (Sigma-Aldrich, Inc.; St. Louis, MO) on coated vesicle formation and virus entry, cells were treated with 6.3 µM of latB for ∼10 min. at 37°C prior to the addition of virus particles.

### Image analysis

Image analysis was performed as previously described [15] with the following modifications. Slidebook 4.2.13 (III) was used to view, scale, and export images for publication. Movies were generated by exporting a series of continuous TIFF files from a Slidebook time-lapse acquisition and compiling the images into a single AVI file using ImageJ (NIH). SigmaPlot 8.0 (SYSTAT; Point Richmond, CA) was used to plot data. Microsoft Excel was used to determine whether data from 2 categories differ in a statistically significant manner using two-tailed Student's t-tests. An automated image analysis application (IMAB) [8] developed within MATLAB (Mathworks; Natick, MA) was used to track the formation of clathrin-coated structures and the internalization of single virus particles. Images were processed for analysis as previously described, and established criteria were used to exclude incomplete endocytic events and events in which pixels assigned to one clathrin structure merged with or split from an adjacent structure [8]. For each cell of interest, the first ∼50 pits lacking virus particles were analyzed in detail to measure the coat lifetime and protein composition (see below). All complete virus uptake events that occurred in these cells were also analyzed in a similar manner. With the exception of internalization events blocked by latB, incomplete internalization events or events truncated by the start/end of image acquisition were not analyzed. Aggregates of virus were identified as objects with fluorescence intensities greater than that of single particles and were excluded from all analyses.

IMAB was used to measure the fluorescence intensity of coat components or virus particles in the following manner. A roughly spherical mask encompassing 69 pixels was centered on the peak fluorescence intensity of the object in all frames that the object was detectable above the local background fluorescence. The contribution of the local background fluorescence was estimated by measuring the average intensity of pixels within a ring of single pixel width that extended from the outer boundary of the object mask. The intensity of the pixels within the object mask was then summed, and the average intensity value of pixels in the local background was subtracted from each pixel within the object mask to estimate the fluorescence contributed by proteins (or dye molecules) within the object of interest.

### Electron microscopy

Purified virus particles were deposited onto carbon-coated copper grids and stained with 2% phosphotungstic acid (PTA) in H_2_O (pH 7.5). To visualize DI-T particles in clathrin-coated pits, BSC1 cells were inoculated with unlabeled DI-T particles using a dose that yielded ∼1000 attached particles per cell after 10 min. Samples were then processed for ultra-thin sectioning as previously described [15], [59]. Electron micrographs of VSV particles in clathrin endocytic structures were obtained from cells infected with VSV for 6 h, where the entry of newly released particles could readily be visualized. Virus particles and ultra-thin sections of cells were viewed using a Tecnai G^2^ Spirit BioTWIN transmission electron microscope (FEI, Hillsboro, OR).

## Supporting Information

Video S1 Clathrin-dependent DI-T internalization. BSC1 cells stably expressing σ2-eGFP (green) were inoculated with DI-T particles, and time-lapse images were acquired at 4 s intervals. A 4.2×4.2 µm^2^ area of the cell surface is shown as in Figure 2B. The video depicts a single DI-T particle (red) that attaches to the plasma membrane and diffuses slowly. A clathrin-coated pit captures the virus particle when a dim adaptor spot colocalizes with the virus signal. The adaptor fluorescence intensity increases as coated pit assembly proceeds, and particle internalization occurs shortly after the adaptor signal peaks. Disappearance of the adaptor signal signifies clathrin uncoating, and the virus-containing vesicle is then transported toward the cell interior.
(0.31 MB AVI)

Video S2 Sequential internalization of single VSV and DI-T particles by the same cell. BSC1 cells stably expressing σ2-eGFP (green) were simultaneously inoculated with wild-type VSV and DI-T particles, and time-lapse images were acquired at 4 s intervals. A 3.5×3.5 µm^2^ area of the cell surface is shown as in Figure 3A. At the outset of imaging (t = 0), one VSV (red) particle and one DI-T particle (blue) are visible on the cell surface. The VSV particle enters first, followed by the DI-T particle.
(0.39 MB AVI)

Video S3 Uptake of a single DI-T particle by a cell expressing eGFP-LCa. BSC1 cells transiently expressing eGFP-LCa were inoculated with DI-T and full-length VSV particles, and images were captured as for Video S2. At the onset of imaging, a DI-T particle is present within a 3.5×3.5 µm^2^ area of the cell surface. Shortly thereafter, the particle briefly colocalizes with a spot of eGFP-LCa but does not enter. As visualized in Figure 3B, a clathrin-coated pit subsequently initiates near the virion, and the particle disappears into the cell after the clathrin signal peaks.
(0.77 MB AVI)

Video S4 Uptake of a single VSV particle by a cell expressing eGFP-LCa. The movie depicts a separate area (of equal size) of the plasma membrane from the same cell that internalized the particle shown in Video S3. A VSV particle (red) attaches to the cell surface, and a clathrin endocytic structure subsequently internalizes the particle (Figure 3D).
(0.77 MB AVI)

Video S5 Cortactin recruitment during conventional clathrin-coated pit formation. Time-lapse images were acquired at 3 s intervals from a cell transiently co-expressing mCherry-LCa (red) and cortactin-eGFP (green) (Figure 5A). A 7.0×7.0 µm^2^ area of the cell surface is shown. Note that the cortactin signal is nearly indistinguishable from the local background during most clathrin endocytic events.
(3.01 MB AVI)

Video S6 Cortactin recruitment during the internalization of a single DI-T particle. BSC1 cells transiently co-expressing mCherry-LCa (red) and cortactin-eGFP (green) for 18 h were inoculated with DI-T particles (blue), and time-lapse images were acquired at 3 s intervals. The video shows the internalization of a single DI-T particle by a clathrin-coated vesicle. The internalization event is centered within a 3.5×3.5 µm^2^ area of the cellular plasma membrane (Figure 5B). The left panel shows an overlay of the 3 channels, and the right panel shows only the cortactin channel displayed in monochrome. Note that the cortactin fluorescence intensity during DI-T entry is nearly indistinguishable from the local background.
(1.42 MB AVI)

Video S7 Cortactin recruitment during the internalization of a wild-type VSV particle. BSC1 cells transiently co-expressing mCherry-LCa (red) and cortactin-eGFP (green) for 18 h were inoculated with VSV particles (blue), and time-lapse images were acquired at 3 s intervals. The video is displayed as described for Video S3 and shows the clathrin-dependent uptake of a single VSV particle in a 3.5×3.5 µm^2^ area of the plasma membrane (Figure 5C). Note the visible burst of cortactin that occurs in the final stages of VSV internalization.
(1.51 MB AVI)

Video S8 BSC1 cells stably expressing σ2-eGFP were treated with 6.3 µM latB for 12 min. Cells were inoculated with DI-T (blue) and VSV (red) particles, and images were acquired at 4 s intervals. The movie shows an 8.8×5.0 µm^2^ area of the plasma membrane. The 2 DI-T particles (the lower particle is already in a coated pit at the onset of imaging) enter in the presence of latB, while the 2 VSV particles are subsequently captured by coated pits but fail to enter the cell (Figure 6A, B).
(4.65 MB AVI)
